# The mechanics of congenital heart disease: from a morphological trait to the functional echocardiographic evaluation

**DOI:** 10.3389/fcvm.2024.1301116

**Published:** 2024-04-08

**Authors:** Martina Avesani, Jolanda Sabatino, Nunzia Borrelli, Irene Cattapan, Isabella Leo, Giulia Pelaia, Sara Moscatelli, Francesco Bianco, PierPaolo Bassareo, Francesco Martino, Benedetta Leonardi, Lilia Oreto, Paolo Guccione, Giovanni Di Salvo

**Affiliations:** ^1^ Division of Paediatric Cardiology, Department of Women’s and Children’s Health, University Hospital of Padua, Padua, Italy; ^2^ Paediatric Cardiology and Congenital Heart Disease Unit, Department of Experimental and Clinical Medicine, Magna Graecia University, Catanzaro, Italy; ^3^ Adult Congenital Heart Disease Unit, A.O. dei Colli, Monaldi Hospital, Naples, Italy; ^4^ Department of Experimental and Clinical Medicine, Magna Graecia University, Catanzaro, Italy; ^5^ Paediatric Unit, Department of Science of Health, Magna Graecia University, Catanzaro, Italy; ^6^ Centre for Inherited Cardiovascular Diseases, Great Ormond Street Hospital, London, United Kingdom; ^7^ Institute of Cardiovascular Sciences, University College London, London, United Kingdom; ^8^ Department of Pediatrics and Congenital Cardiac Surgery and Cardiology, Ospedali Riuniti, Ancona, Italy; ^9^ Department of Cardiology, Mater Misericordiae University Hospital and Our Lady’s Children’s Hospital, University College of Dublin, Crumlin, Ireland; ^10^ Department of Internal Clinical, Anesthesiological and Cardiovascular Sciences, La Sapienza University, Rome, Italy; ^11^ Department of Pediatric Cardiology, Cardiac Surgery and Heart Lung Transplantation, Bambino Gesu Children’s Hospital and Research Institute, IRCCS, Rome, Italy; ^12^ Department of Clinical and Experimental Medicine, University of Messina, Messina, Italy; ^13^ Mediterranean Pediatric Cardiology Center, Bambino Gesù Children’s Hospital, Taormina, Italy

**Keywords:** cardiac mechanics, congenital heart diseases, advanced echocardiography, speckle tracking, three-dimensional echocardiography

## Abstract

Advances in pediatric cardiac surgery have resulted in a recent growing epidemic of children and young adults with congenital heart diseases (CHDs). In these patients, congenital defects themselves, surgical operations and remaining lesions may alter cardiac anatomy and impact the mechanical performance of both ventricles. Cardiac function significantly influences outcomes in CHDs, necessitating regular patient follow-up to detect clinical changes and relevant risk factors. Echocardiography remains the primary imaging method for CHDs, but clinicians must understand patients' unique anatomies as different CHDs exhibit distinct anatomical characteristics affecting cardiac mechanics. Additionally, the use of myocardial deformation imaging and 3D echocardiography has gained popularity for enhanced assessment of cardiac function and anatomy. This paper discusses the role of echocardiography in evaluating cardiac mechanics in most significant CHDs, particularly its ability to accommodate and interpret the inherent anatomical substrate in these conditions.

## 1 Introduction

Cardiac function in adults is assessed using echocardiography, following specific guidelines and parameters. In children, the wide variety of congenital heart diseases (CHDs) has led the guidelines for echocardiographic evaluation to be more focused on morphological rather than functional aspects (1).

However, advances in cardiac surgery have led to a recent growing epidemic of children and young adults with CHDs, where congenital defects themselves, surgical operations and residual lesions can alter cardiac anatomy and affect the mechanical efficiency of both ventricles differently. Cardiac function is an important determinant of outcomes in CHDs (2); thus, it is mandatory to adequately follow up patients to identify changes in their clinical status and relevant parameters for risk stratification (3). Echocardiography continues to be the primary imaging technique in CHDs. When performing an echocardiography, clinicians should know patients' anatomy properly, since different CHDs have different anatomical peculiarities that may alter cardiac mechanics. In addition to standard measurements, the use of myocardial deformation imaging and three dimensional echocardiography (3D) has become popular to better assess cardiac function and anatomy (3, 4).

In this paper, we will discuss the role of echocardiography in assessing cardiac mechanics in most significant congenital heart diseases, with a keen focus on how it accommodates and interprets anatomical features inherent to these conditions.

## 2 Atrial septal defects

### 2.1 Anatomy and pathophysiology

The pathophysiological feature of an atrial septal defects (ASD) is known to be right heart volume overload due to left-to-right shunting. When congenital ASDs are repaired during early childhood, patients tend to have a life expectancy similar to that of the general population. While the right ventricular (RV) volume overload and the increased end-diastolic dimensions are well tolerated for an extended period, they ultimately lead to diminished RV function, hypokinesia, and heart failure, resulting in increased morbidity, including arrhythmias, and higher mortality rates.

### 2.2 Echocardiographic assessment

A previous study by Jategaonkar et al. (5) found that people with chronic RV volume overload caused by an ASD have not only higher tricuspid annular plane systolic excursion (TAPSE) values but also higher myocardial strain values compared to healthy populations of the same age. These observations primarily rely on changes in the lateral segments, particularly the mid and apical lateral segments of the RV wall.

Moreover, in a proof-of-concept study by Wu et al. (6) a non-invasive analysis of right ventricular myocardial work (RVMW) was conducted in a cohort of 29 individuals with atrial septal defects. The results revealed that the ASD patients with RV volume overload exhibited significantly higher values for the following parameters: right ventricular global work index (RVGWI), right ventricular global constructive work (RVGCW), and right ventricular global wasted work (RVGWW) in comparison to the control group. However, there was no statistically significant difference observed in right ventricular global work efficiency (RVGWE) between the two groups.

The increased strain values due to RV overload tend to return to normal levels after both percutaneous and surgical ASD closure, according to Jategaonkar (5). Other studies showed nearly normalization of RV strain patterns in patients who underwent percutaneous closure of the atrial defect, while in patients who received surgical correction right ventricular strain remained altered even after 6 months from surgery (7). Moreover, in a study by Di Salvo. et al, atrial strain itself seemed to be altered in patients who received surgical closure of the defect, while peak systolic strain doesn't seem to differ from healthy control in the device closure group (8).In details, speckle tracking analysis across the device showed almost no deformation on the ASD occluder as if strain imaging was not influenced by global heart motion and tethering from adjacent segments, while myocardial velocities failed to significantly discriminate between this noncontracting structure and the normal atrial wall (9).

Regarding the long-term follow-up after ASD closure, a study by Menting et al. (10) demonstrated that, even in patients who underwent ASD closure during childhood, a noteworthy reduction in the right ventricular lateral wall longitudinal strain can be observed 35 years after the closure procedure, compared with the healthy population. This reduction is likely attributed to preoperative chronic volume overload or may be related to the surgical closure technique. One possible explanation is that in the remodelled RVs of ASD patients, the apical segment appears to have a straighter configuration than in healthy subjects, resulting in higher wall stress in these particular locations.

## 3 Ventricular septal defects

### 3.1 Anatomy and pathophysiology

Patients who have large ventricular septal defects (VSDs) leading to excessive blood flow in the lungs demonstrate an enlarged left ventricular end-diastolic diameter (LVEDD) due to the substantial overload of blood volume in the left ventricle. These patients may exhibit differences in contractility in comparison to individuals with hearts that have normal structural characteristics. Although several studies have indicated the presence of systolic dysfunction in individuals with VSDs, the replication of these findings has been inconsistent. On the other hand, it is conceivable that contractility may be augmented in specific patients as a compensatory mechanism to counterbalance the decrease in systemic output resulting from excessive pulmonary circulation. This observation implies that there could potentially exist variations in contractility among individual subjects with VSDs.

### 3.2 Echocardiographic assessment

According to a study conducted by Penk et al. (11), the evaluation of contractility through the measurement of left ventricular longitudinal strain and strain rate did not yield statistically significant distinctions between patients with VSDs who were recommended for surgical intervention and healthy children in a baseline state.

Similarly, Kotby et al. (12) found no statistically significant differences in the mean global peak longitudinal systolic strain between patients and controls, asymptomatic groups and groups with symptoms, and people with dilated left ventricles (LV) and those without LV dilation. This suggests that keeping systolic function in people with volume overload can be important enough to go unnoticed even when using 2D speckle tracking echocardiography (STE)-derived longitudinal strain, a method that is known for being able to find subtle ventricular dysfunction. To figure out what's going on, researchers are looking at eccentric hypertrophy and myofibril remodeling, which happen when the ventricles are overloaded with blood and help keep systolic function going. Magee et al. (13) conducted a study employing a unique methodology and found results consistent with those of previous studies. In particular, they saw that older children and adults with VSD and moderate left-to-right shunting had either the same amount of systolic function or more systolic function than their control groups.

Favorable long-term outcomes are observed in the surgical correction of VSDs during infancy. In the present study, Kwon et al. (14) examined infants and neonates who had symptomatic VSD requiring surgical intervention. The authors observed that post-surgery, there was an anomalous movement of the ventricular septum, which coincided with a decrease in preload. The observed changes in septal motion resulted in a subsequent decline in left ventricular torsion during the immediate postoperative period.

Even though ventricular systolic dysfunction can happen right after surgery, it is important to note that most patients' left ventricular mechanics return within the first year.

## 4 Left ventricular outflow tract obstructions

### 4.1 Anatomy and pathophysiology

Coarctation of the aorta (CoA) is characterized by a narrowing of the aorta, typically found just beyond the left subclavian artery and near the point where the ductus arteriosus attaches, sometimes associated with transverse arch hypoplasia. While CoA has been considered simply a focal stenosis, it is now established that it is a complex disease of vasculature (15).

Indeed, mechanical obstruction at the aortic isthmus, potentially associated with others left-side lesions, and vascular dysfunction lead in the long term to increased LV pressure and wall stress (16). This, in turn, causes the development of compensatory concentric left ventricular hypertrophy (LVH).

Surgical correction is essential to remove the stenosis, but LV function may remain subnormal in many patients even years after surgery. Also, patients have an increased aortic stiffness and a reduced aortic distensibility regardless of the stenosis, and often develop systemic hypertension which, in turn, may provoke LVH and, ultimately, systolic and diastolic dysfunction (17).

### 4.2 Echocardiographic assessment

Although the diagnosis of CoA is still the fetal cardiologist's Achilles heel, some data suggest that LV remodeling starts during fetal life. Data from Soveral et al. found that, compared to controls, foetuses with CoA had a right dominance due to smaller LV cavities, and to an abnormally elongated LV. They also found a preserved LV function and thickness and hypothesized that volume redistribution prevents LV pressure increase during fetal life. After birth, the LV tries to adapt to the acute increase of volume through left-side cavities with a more globular shape and augmented filling velocities (18).

A study investigating diastolic and systolic performance with Doppler Tissue Imaging (TDI) in patients who underwent CoA repair in neonatal period vs. later repair and controls found that diastolic and systolic performance were altered in both groups of patients preoperatively. An improvement of systolic and diastolic performance was noticed postoperatively both in neonate and infants; however, diastolic function seems to remain impaired in both groups compared to controls after the first postoperative year. By contrast, systolic performance was found to be more persistently altered in patients who underwent surgical repair in the neonatal period, suggesting a higher hemodynamic stress in this group before surgical correction (19).

Avendaño-Pérez et al. showed that adult patients with unrerpaied CoA had LVH, an increased LV mass, lower values of LV EF and GLS compared to controls, and that values of myocardial deformation were inversely related to LV mass. Also, GLS was reduced in patients with preserved EF, confirming the ability of this technique in detecting subclinical dysfunction (20).

Indeed, regional longitudinal systolic myocardial deformations as assessed by strain rate (SR) were significantly reduced in a large sample of young normotensive patients years after successful CoA repair in the presence of a normal/increased LVEF. Also, the degree of longitudinal SR impairment was correlated with age at repair and aortic stiffness. The increased aortic stiffness in these patients demonstrates that, despite a successful repair, surgery cannot change the intrinsic abnormalities in vascular structure and function (21).

**Figure 1:**
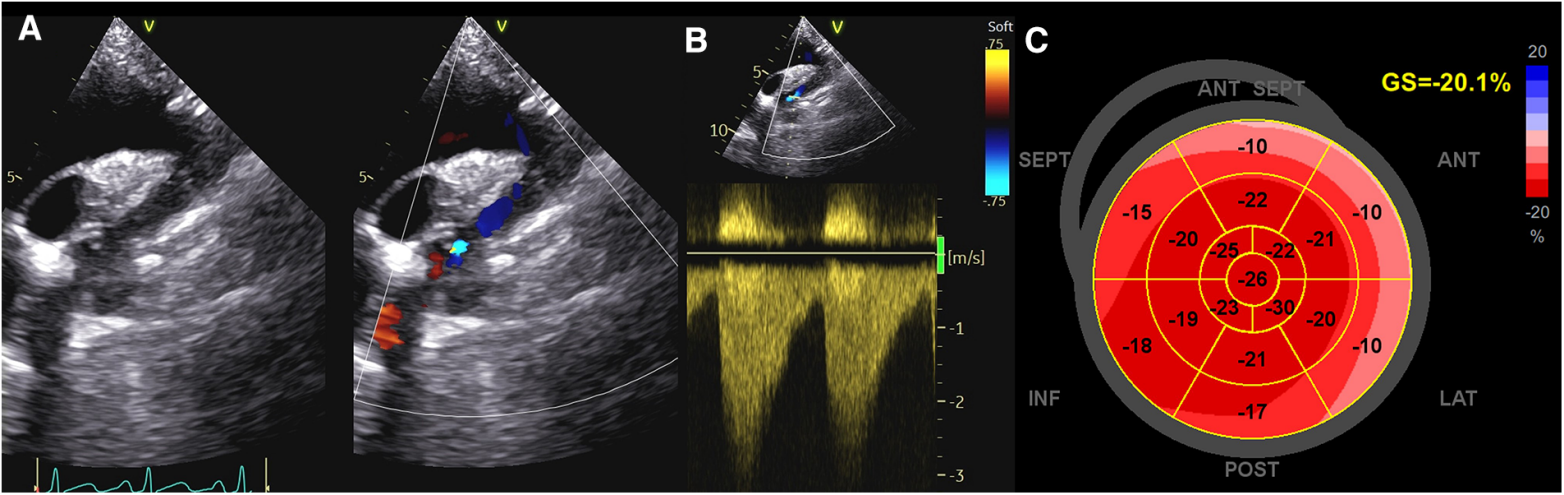
Evaluation of systolic function in a patient with coarctation of the aorta. (**A,B**) CoA as assessed by Color and CW Doppler; (**C**) GLS values in the same patient. As noticed, strain values are reduced in basal segments, which are impaired also in hypertensive patients. CoA, coarctation of the aorta; CW, continuous Doppler; GLS, global longitudinal strain.

In addition, a reduction of longitudinal strain values of basal septal segment (≤−16.6%) and of LV twist were found to be the best predictors of masked hypertension in adolescents with repaired CoA. The impairment of basal segment function, which is impaired also in hypertensive heart disease, could probably be explained by the increased regional stress on the basal subendocardial longitudinal fibers at that level (22) (Figure 1).

Lastly, left atrial (LA) function, assessed by STE as reservoir phase, was found to be frequently impaired in adolescents and young adults years after CoA repair, especially in those with altered aortic arch geometries (i.e gothic aortic arch) and in those with atrial arrhythmia and stroke (23). These represent some of the most feared morbidities after CoA repair. Thus, investigating LA reservoir function, an early indicator of both LA and LV function in conditions of elevated afterload, could be useful to identify patients at higher risk of morbidity.

## 5 Tetralogy of Fallot

### 5.1 Anatomy and pathophysiology

Tetralogy of Fallot (TOF) is most likely the best example of the success of paediatric cardiology and cardiac surgery, but success is not a miracle, and as a result, the survival rate among adults remains significantly lower than that of the general population. This discrepancy is primarily attributed to a heightened occurrence of heart failure (HF), ventricular arrhythmias, and sudden cardiac death, with heart failure being the primary cause of mortality in adult patients with repaired TOF (rTOF) (24, 25).

The underlying mechanisms leading to cardiac dysfunction in Tetralogy of Fallot seem to involve an adverse biventricular response and (mal)adaptation to multiple stressors such as RV pressure and/or volume-overload, surgery, myocardial fibrosis, and electro-mechanical dyssynchrony (5). Genetics and acquired mechanisms contribute to this biventricular response, often termed “remodeling”, starting from fetal life, and continuing into adulthood (25).

Histological analysis of myocardial biopsies from the RV at the time of surgical repair showed increased cardiomyocyte diameters and higher interstitial fibrosis in both ventricles compared to normal reference values (26). These changes were particularly prominent in older patients with longer exposure to cyanosis and pressure overload, as well as in individuals experiencing myocardial dysfunction and ventricular arrhythmias (27). A study comparing the myocardial histopathology of adults with TOF who had late repairs and those who didn't showed that, at the same age of death, the rate of hypertrophy and fibrosis progression in the two ventricles was the same in both groups, even though the repaired hearts had less of both (28).

Furthermore, there are differences in ventricular myocardial architecture between individuals with TOF and those with normal hearts. In people with TOF, the RV sub-epicardial fibers are more angled, and there is a clear layer of circumferential fibers in the middle, especially at the level of the sub-pulmonary infundibulum. These features have been observed in both adult patients with rTOF and in children before undergoing surgery (29). This highlights the unique pathophysiology and complexity of TOF, where various loading conditions and surgical interventions contribute to cardiac remodeling.

Lastly, it is essential to acknowledge the interdependence between the LV and the RV, as they share myofibers, the septum, coronary blood supply, and the pericardium (30). Thus, it is logical that the function of one ventricle influences and is influenced by the other one.

### 5.2 Echocardiographic assessment

With 2D echocardiography, it is difficult to properly assess the RV, but advancements in 3D echocardiography and speckle tracking imaging provide valuable new information for evaluating ventricular function in TOF patients. Recent 3D echocardiography data shows that the shape of the RV curvature changes during the cardiac cycle in repaired TOF patients with severe pulmonary regurgitation (PR). Indeed, compared to control subjects, those with rTOF and severe PR exhibit a flatter RV-free wall, with a tilt of the tricuspid annulus, a rounded apex, and a more convex right ventricular outflow tract (RVOT) and interventricular septum. Furthermore, their mid-RV free wall and interventricular septum become less convex from end-diastole to end-systole, while apical-free wall convexity increases during end-systole (31). These geometrical changes in wall curvature are expected to alter wall stress and regional myocardial remodeling.

Other studies have shown that people with rTOF and PR have lower RV global longitudinal and circumferential strains (32, 33). The longitudinal component of the RV free wall seems to be the most affected (33).

Right bundle branch block (RBBB) is common after TOF repair and can be caused by damage to the heart at different stages (34). How much the ventricular activation sequence is delayed depends on where the damage is. This delayed activation of the RV free wall may cause significant electrical dyssynchrony and this may contribute to mechanical inefficiency and dysfunction (35).

Hui et al. conducted a study with STE which revealed that a right-sided septal flash, a marker of RV intraventricular electro-mechanical dyssynchrony, with concomitant early prestretch and late contraction of the RV basal lateral wall is common in rTOF patients, and intra-RV delay is more prominent in individuals with higher RV volumes (36).

Also, reduced RV deformation in association with RV dyssynchrony and decreased exercise tolerance have been demonstrated in asymptomatic rTOF children (37), suggesting a potential role of resynchronization therapy in improving RV dysfunction.

Lastly, in rTOF patients, RV diastolic function is also impaired. Different levels of dysfunction have been reported, but the assessment of RV diastolic function with standard echocardiographic is still a challenge (38). Promising data are emerging about the role of right atrial strain to investigate RV diastolic function and to identify patients at higher risk of adverse events (39, 40).

In addition to right ventricular dysfunction, a reduction in longitudinal, radial, and circumferential strain has been demonstrated also for the LV, despite a preserved ejection fraction. The LV radial component seems to be more affected than the other components (41). Recent study demonstrated that in rTOF patients LV torsion, a main determinant of LV mechanics, is impaired. This impairment is characterized by a reversed (counterclockwise) basal rotation along with a compensatory increase in apical rotation at a younger age (41, 42), which becomes impaired later in life (43). Changes in rotational mechanics may indicate that the disease is in a more advanced stage and are linked to events like death, heart failure, arrhythmia, reintervention, or hospitalization for cardiac reasons (44). Notably, RV strain was identified as the sole predictor of reversed counter-clockwise LV basal rotation, suggesting that RV dysfunction, rather than RV dilatation alone, could potentially play a crucial role in abnormal LV rotation and mechanics (41).

## 6 D-transposition of great arteries

### 6.1 Anatomy and pathophysiology

In D-transposition of great arteries (D-TGA), during foetal life and before surgical correction, the right ventricle acts as the systemic pump, while the left ventricle, situated under the pulmonary circulation, experiences low systolic pressure. Cardiac surgery is usually performed shortly (<7–10 days) after birth. This is mainly because there is concern about the left ventricle's ability to cope with the abrupt transition to the pressure load of the systemic circulation and to avoid the development of pulmonary vascular disease.

The arterial switch operation (ASO) is currently the standard surgical procedure to treat D-TGA, with remarkable outcomes in reducing mortality rates and the necessity for further interventions (45). However, coronary artery abnormalities and reimplantation, reduced coronary flow reserve, intimal proliferation, the development of significant neo-aortic regurgitation, and RVOT obstruction may impair cardiac function in the long term (46, 47). Also, approximately 10% to 20% of patients who underwent ASO has a gothic aortic arch (GAA), a particular anatomical variation distinguished by an elevated, slender, and elongated aortic arch (48). The pathogenesis of this variation is not fully understood, but an altered geometry and tension of the aorta following ASO as well as genetic and developmental factors may play a role.

### 6.2 Echocardiographic assessment

Few studies have investigated cardiac mechanics and morphology in foetuses with D-TGA. Reduced global and regional RV longitudinal systolic peak velocity, strain, and strain rate, as well as a more globular RV shape compared to controls, were found (49, 50). After birth, this morphological aspect persists before surgical correction, while RV function is usually normal or mildly impaired (50, 51).

When evaluated using conventional echocardiographic parameters after ASO, LV systolic function is typically within the range of normal (52). However, LV functional abnormalities have been shown by some studies both under pharmacological stress (53) and at rest (54), especially in patients with variant coronary arterial anatomy (55). Also, a significant reduction in the longitudinal systolic myocardial deformation with normal circumferential deformation and torsion has been found in asymptomatic patients years after ASO, and age at surgery was the only variable significantly correlated with global LV longitudinal systolic deformation (56). These findings altogether may suggest that: (1) an older age at the time of surgery might be linked to a decrease in LV mass and an underdeveloped left anterior descending coronary artery, which supplies the larger amount of myocardial mass; (2) consequently, these factors could lead to abnormal global longitudinal deformation, which represents an early marker of cardiac dysfunction. (3) Normal torsion might serve as a compensatory mechanism to preserve LV function.

Lastly, the presence of a GAA can change the dynamics of blood flow, with variations in shear stress distribution along the aortic wall that may cause arterial stiffness, vascular remodeling, and, ultimately, cardiac dysfunction. Studies investigating myocardial strain in patients with GAA after ASO revealed changes in strain values and distribution patterns, particularly in the basal segments of the LV (57). These alterations may be attributed to increased wall stress in those segments, as explained by Laplace's law.

## 7 Ebstein's anomaly

### 7.1 Anatomy and pathophysiology

Ebstein's Anomaly (EA) is a rare CHD involving the tricuspid valve and the RV. It is caused by a failure in the delamination process during embryologic development which results in septal and posterior leaflets typically adherent to the underlying myocardium and in a dysplastic anterior leaflet, with varying degrees of redundancy and fenestration (58). In EA, the RV can be divided into two distinct regions (58, 59). The inlet portion becomes in fact functionally integrated into the right atrium due to the TV malformation. This “atrialised” portion is usually thin, dilated and poorly contractile, serving as a passive reservoir during atrial contraction. The number of RV myocardial fibers is decreased, contributing to thinning and decreased myocardial efficiency (60). The remaining “functional” RV (fRV) is also dilated, with concomitant tricuspid annular dilatation and various degree of regurgitation. The RV myocardial cells are normally organized in a specific structure; the epicardial fibers are in fact obliques and continuous with the LV ones, the mid-wall circumferential layer is poorly developed, and the endocardial ones are longitudinally oriented (61). This explains why RV contraction usually relies more on longitudinal shortening than circumferential deformation (61). Abnormal loading conditions can change this asset, with a greater contribution of circumferential and radial shortening components to global RV ejection (61).

### 7.2 Echocardiographic assessment

**Figure 2:**
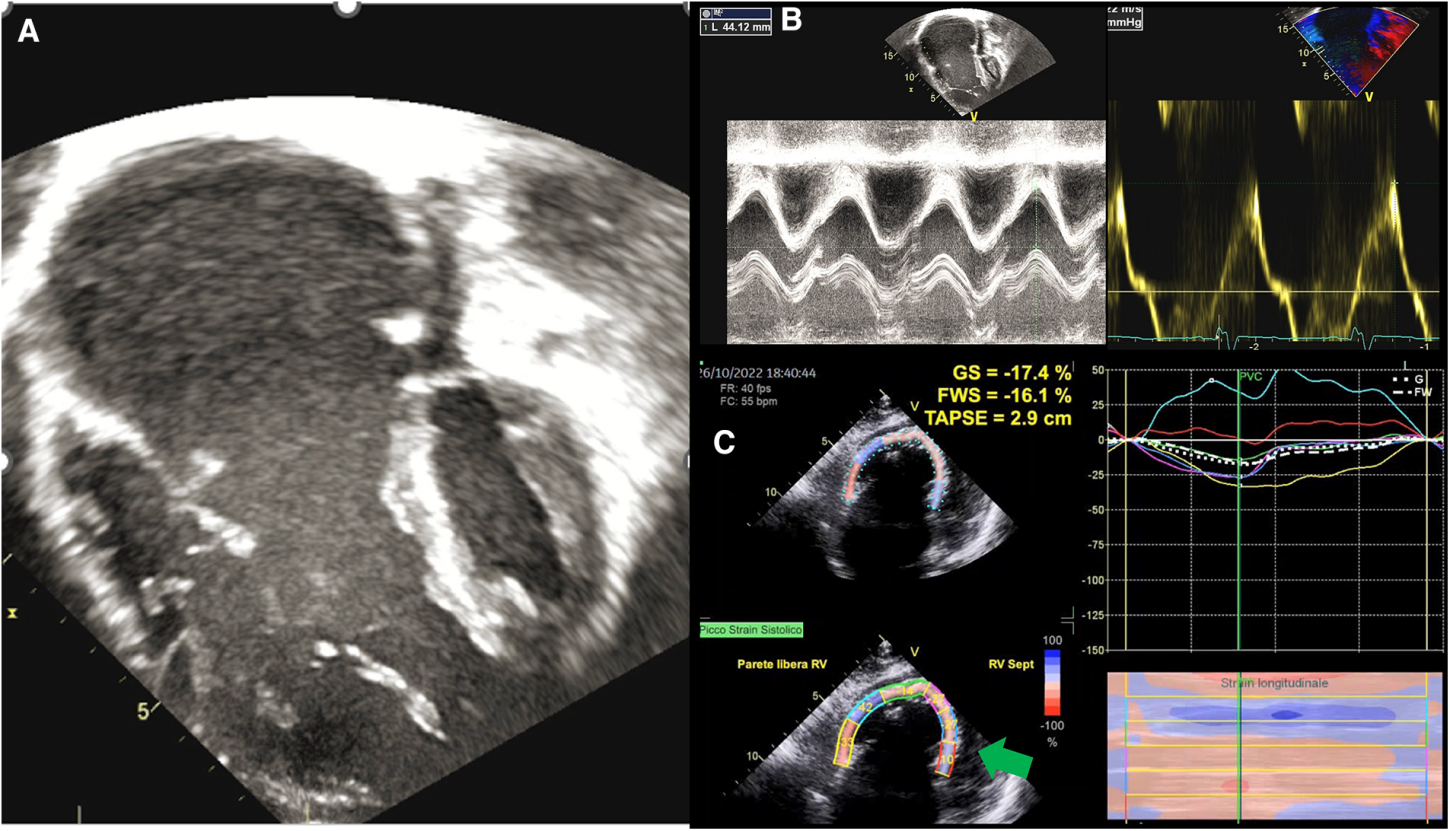
Functional assessment in ebstein anomaly. (**A**) EA as visualized by 2DE; (**B**) standard echocardiographic parameters to assess RV function, such as TAPSE an S’ wave, are increased in EA because of volume overload; (**C**) GLS analysis shows that the RV in EA is “atrialised” not only morphologically, but also functionally, as basal and medial septal strain values (green arrows) are positive in systole, thus behaving as an atrium. EA, ebstein anomaly; 2DE, two-dimensional echocardiography; TAPSE, tricuspid annular plane systolic excursion; GLS, global longitudinal strain; RV, right ventricle.

The evaluation of RV volumes and function by echocardiography is often difficult due to the retrosternal position of the RV and its complex geometry (62). This is particularly true in patients with EA, where the morphological abnormalities of the RV and the altered loading conditions (i.e., volume overload due to tricuspid regurgitation) further challenge this assessment (Figure 2). Most of the conventional echo parameters used to assess RV function may therefore be unreliable (63). In a small study of 16 EA's patients, measurement of fractional area change (FAC) was unfeasible due to difficulties in detecting the endocardial border (64). However, in another study including only young and unrepaired EA patients (*n* = 50) FAC assessment was feasible in the whole cohort (65). The authors found that this functional index also had prognostic significance, being the only predictor of progressive disease along with right atrium peak systolic strain (RA-PALS) (65). The RV-FAC is the expression of the contraction of the obliquely oriented fibers. This data confirms that the longitudinal function may be compromised early in EA patients, with an increased compensatory circumferential deformation reflected by FAC (65).

Kühn et al. compared TAPSE, tissue Doppler myocardial velocities (peak systolic myocardial velocity, s', and isovolumic acceleration) and 2D strain and strain rate measures with cardiac magnetic resonance (CMR)-derived EF. Of the six parameters investigated, only 2D global longitudinal strain (GLS) had a good correlation with CMR-derived RVEF. Of note, it was also the only parameter showing a good inter- and intra- observer variability in their study (63). Results of a more recent study enrolling 620 patients with EA, confirmed the importance of implementing the use of RV GLS in risk stratification of EA patients. RV GLS was in fact identified in this larger cohort as an independent predictor of all-cause mortality and cardiovascular mortality, with superior prognostic power than RV FAC, RV s', or TAPSE (66). In particular, RV s' and TAPSE did not correlate at all with clinical outcomes, perhaps reflecting more segmental rather than global systolic function (66).

Data coming from STE can also be helpful to understand how mechanical dyssynchrony can impact RV function. Intra- RV conduction delay can be assessed using the standard deviation of time to peak shortening among the fRV segments. Patients with EA have mechanical dispersion, as demonstrated by the presence of abnormal early functional RV septal activation, RV lateral wall prestretch/late contraction, postsystolic shortening and increased intra-RV delay measured by STE (67). This is clinically relevant, as associated with fRV remodeling, dysfunction and impaired exercise capacity (67).

Although the exact underlying mechanisms are not yet fully understood, LV dysfunction has been described in up to 50% of patients with EA (68). The dilatation of the RV causing septal bowing and compression of the LV can be in part responsible for LV impairment, although it is unlikely to represent the only mechanism (69, 70). Along with patients with overt LV dysfunction, there is a proportion of patients with normal EF but small volumes and low stroke volumes (71). These patients have worse right heart dysfunction, higher diastolic interventricular dependence (higher diastolic eccentric index), and therefore reduced LV pre-load and stroke volumes (71). The reduced LV end-diastolic volume provides a normal LVEF value even in the presence of LV dysfunction. The relatively load-independent GLS can unmask this occult dysfunction and be used as an early marker of impaired function (71). A reduced absolute LV GLS, but not LVEF, was an independent predictor of death or cardiac transplantation and was associated with suboptimal postoperative LV reverse remodeling (72, 73). The presence of mechanical dyssynchrony can further contribute to LV dysfunction and should be assessed in these patients. An increased circumferential strain dyssynchrony index calculated as (peak segmental average−peak global average)/peak segmental average has been observed in neonates with EA and tricuspid valve dysplasia and is associated with an increased risk of mortality (73).

## 8 Univentricular heart

### 8.1 Anatomy and pathophysiology

The term “univentricular heart” refers to various complex congenital heart conditions where both atria primarily connect to a single functional ventricle, preventing the possibility of biventricular repair. These defects can involve either a single right ventricle or a single left ventricle. Treatment typically involves a staged approach, including a neonatal Norwood stage I procedure, a Glenn procedure or Norwood stage II around 6 months of age, and Fontan completion surgery around 4 years of age (74).

From infancy to Fontan palliation, univentricular hearts face unique load conditions (75). The Norwood stage I procedure causes systemic and pulmonary circulation to work in parallel, leading to chronic volume overload in the single ventricle, causing it to enlarge and gain a spherical shape. After the Glenn procedure, venous blood from the upper body must pass through the lung to reach the heart, reducing ventricular volume load and potentially improving atrioventricular valve and myocardial function. Finally, with Fontan surgical completion, the systemic venous blood flow results completely redirected, and the ventricular volume load is significantly reduced.

Moreover, in the presence of a volume-contracted condition and preserved myocardial mass, the single ventricular mass/volume ratio results increased, with consequent high myocardial wall stiffness and risk of diastolic dysfunction. In this setting, further factors (e.g., residual coarctation of aorta, subaortic stenosis, ventricular dyssynchrony, myocardial scars, systolic ventricular dysfunction) may worsen myocardial relaxation and be responsible for diastolic dysfunction (76). However, long-term Fontan circulation can lead to isolated diastolic dysfunction, further impacting clinical prognosis (77). Multiple factors, including reduced cardiac output reserve, renal and ventilatory dysfunction, and autonomic nervous system activation, may contribute to heart failure with preserved ejection fraction (HfpEF) (78).

Univentricular hearts differ from biventricular hearts also in terms of myo-architecture. Indeed, the single right ventricle has a thicker circumferential layer and reduced longitudinal layer, leading to more circumferential contraction (79). Moreover, hypoplastic left hearts exhibit myocardial fibers disarray with variable myocyte size (80) and fibrosis, contributing to systolic and diastolic dysfunction (75).

Finally, ventricular interdependence is lost in the presence of single ventricle physiology. However, in some studies (81–83) in patients with hypoplastic left heart syndrome, the presence of large remnants of the LV has been associated with impaired single RV function and worse outcomes, but other studies failed to confirm this association (84). A recent Swedish study with 20 years of follow-up has demonstrated that a thickened, globular left ventricle with endocardial fibroelastosis and aortic atresia-mitral stenosis subtype represent morphological risk factors for worse outcomes in patients with hypoplastic left heart syndrome (85).

### 8.2 Echocardiographic assessment

**Figure 3:**
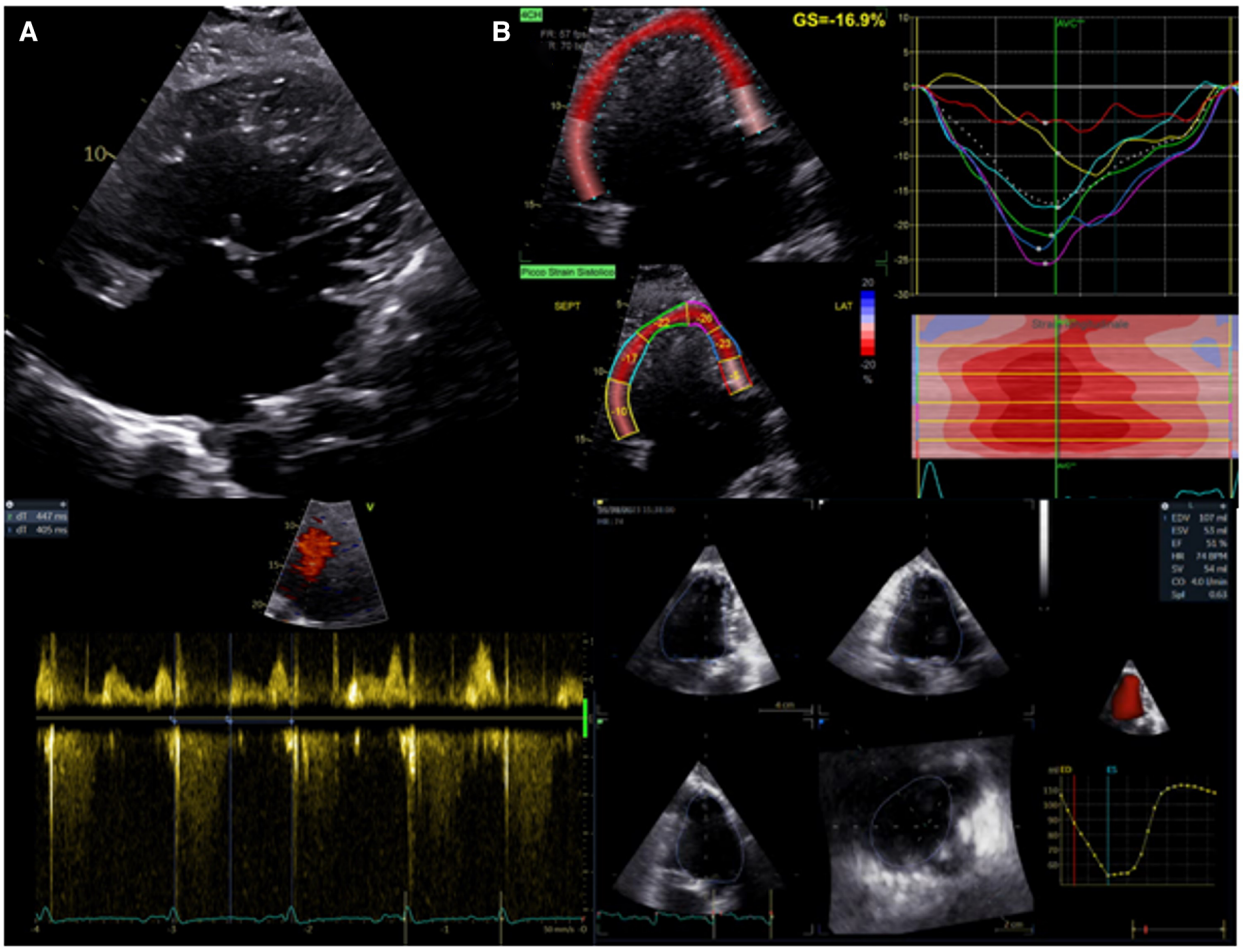
Evaluation of systolic function in patients with univentricular heart after fontan procedure. (**A**) Upper panel: apical 2D view. Lower left panel: atrioventricular systolic to diastolic duration ratio. (**B**) Upper panel: univentricular speckle tracking analysis. Lower right panel: 3D ejection fraction evaluation.

In the presence of a univentricular heart with left morphology, the most common approach to estimate systolic function is the biplane Simpson's method. Even though this method is load-dependent and influenced by geometry, it has a moderate correlation with MRI measurements and reasonable reliability (86). Even more challenging can be the evaluation of a morphological right single ventricle. FAC is a simple geometric method, but its accuracy can be limited by the complex anatomical geometry and the high inter and intra-observer variability (87). Of note, these measurements have no precise normal values for univentricular hearts, but they are valuable when evaluated in the longitudinal follow-up. The atrioventricular systolic to diastolic duration ratio has been used to evaluate ventricular performance without anatomical assumptions. Cordina et al. (88) demonstrated that a value >1.1 independently predicts mortality in adult patients with Fontan circulation. STE has emerged as a valid and reliable method for assessing single-ventricle function without geometric assumptions or the effect of acute preload (89). When the GLS is evaluated serially in the follow-up of children with hypoplastic left heart syndrome, it has been demonstrated to predict ventricular dysfunction leading to need for transplantation or death (87). A recent study (90) demonstrated the different value of longitudinal strain related to the different univentricular morphology. Single right heart has shown lower longitudinal strain than biventricular and single left heart morphology, underscoring a worse morbidity associated with this subgroup. STE has also been used to demonstrate a dyssynchronous pattern in patients with Fontan circulation. The dyssynchronous segments showed an early shortening (“flash”), followed by a systolic stretching; while segments with conduction delay showed early stretching, followed by delayed contraction. A recent study (91) demonstrated this dyssynchronous pattern was associated with a worse outcome in patients with Fontan circulation. 3D echocardiography allows the evaluation of univentricular dimensions and function without geometrical assumptions. The evaluation of three-dimensional ejection fraction and volume appears feasible with a good correlation with CMR, although it is noteworthy to underscore the underestimation of measurements in comparison with CMR (92) (Figure 3).

Even in the presence of a preserved single ventricle systolic function, diastolic dysfunction may be commonly present in patients with Fontan circulation. The E/e' ratio has been demonstrated to poorly correlate with invasive filling pressure and should not be used as an index of diastolic function in patients with Fontan circulation (93). The presence of a considerable lengthening (>28 ms) of the pulmonary venous atrial reverse flow relative to atrial forward flow time into the ventricle, raises the possibility of a high filling pressure of the single ventricle. A short atrioventricular valve's inflow deceleration time has also been linked with diastolic dysfunction (84). Recently, Chowdhury et al. (78), demonstrated E:e'/end-diastolic volume ratio with a cutoff value of 0.26 ml-1 reasonably identifies HFpEF in paediatric patients with Fontan circulation.

## 9 Systemic RV in biventricular physiology

### 9.1 Anatomy and pathophysiology

It is estimated that systemic RV (sRV) account for 10 to 12% of all CHDs (94). Systemic right ventricles in biventricular physiology are commonly encountered in TGA with previous atrial switch repair (Senning or Mustard operation) and Congenital Corrected Transposition of Great Arteries (ccTGA). Despite all the adaptational mechanisms though, the RV seems not to be able to sustain systemic circulation in the long run, as most patients develop ventricular dysfunction in time (95). Nevertheless, this process is not yet fully understood (96) In fact, there are several confounders that can have an impact in determining systemic RV failure, like the presence of associated lesions (like Ebstein anomaly in ccTGA), intrinsic or acquired conduction abnormalities and previous surgeries.

In terms of physiology, considering its high surface/volume ratio, the thin wall and the high compliance, the normal RV is well suited for managing large volumes of blood and changes in preload and, vice versa, is poorly tolerant of acute changes in afterload (97). Myocardial fibers architecture supports the role of the right ventricle as a “volume” chamber, rather than a “pressure” chamber. Unlike the LV, in fact, the right one is composed only of two layers of myofibers. The sub-epicardial layer accounts for approximately 25% of wall thickness and it is formed by predominantly circumferential aggregates, while the subendocardial layer is composed of predominantly longitudinally oriented fibers (98). This peculiar arrangement results in the typical longitudinal-peristaltic contraction pattern of the sub-pulmonary RV (99, 100).

When an RV is in a systemic position, it faces increased afterload and therefore it undergoes some morphological and functional adaptations. Indeed, it shows hypertrophy and a change in myocardial fiber orientation with a higher proportion of circumferentially oriented elements (101, 102). Since circumferential shortening is one of the main features of normal left ventricular mechanics, this appears to be an adaptive mechanism of the sRV to the increased afterload, although the virtual absence of the torsional deformation may represent a potential cause of systolic dysfunction (103). Hypertrophy itself seems to be detrimental in the long term as it is associated with increased oxygen demand as well as reduced myocardial capillary density, leading to potential supply/demand mismatch (104), and prolonged microvascular ischemia can then lead to right ventricular fibrosis (105, 106).

In addition, tricuspid regurgitation can be one of the causes and one of the effects of systemic right ventricular dysfunction as well (107). Tricuspid valve, in fact, can be congenitally altered and mechanisms like annulus dilatation and septal leaflet traction due to right-to-left septal shift can worsen valve regurgitation. Those two mechanisms though, are also the results of progressive ventricular dilatation, which creates a detrimental loop in between regurgitation and dysfunction.

Finally, conduction disorders and heart blocks that require pacing can be very common comorbidities that lead to ventricular dyssynchrony and enhance ventricular dysfunction (108).

### 9.2 Echocardiographic assessment

**Figure 4:**
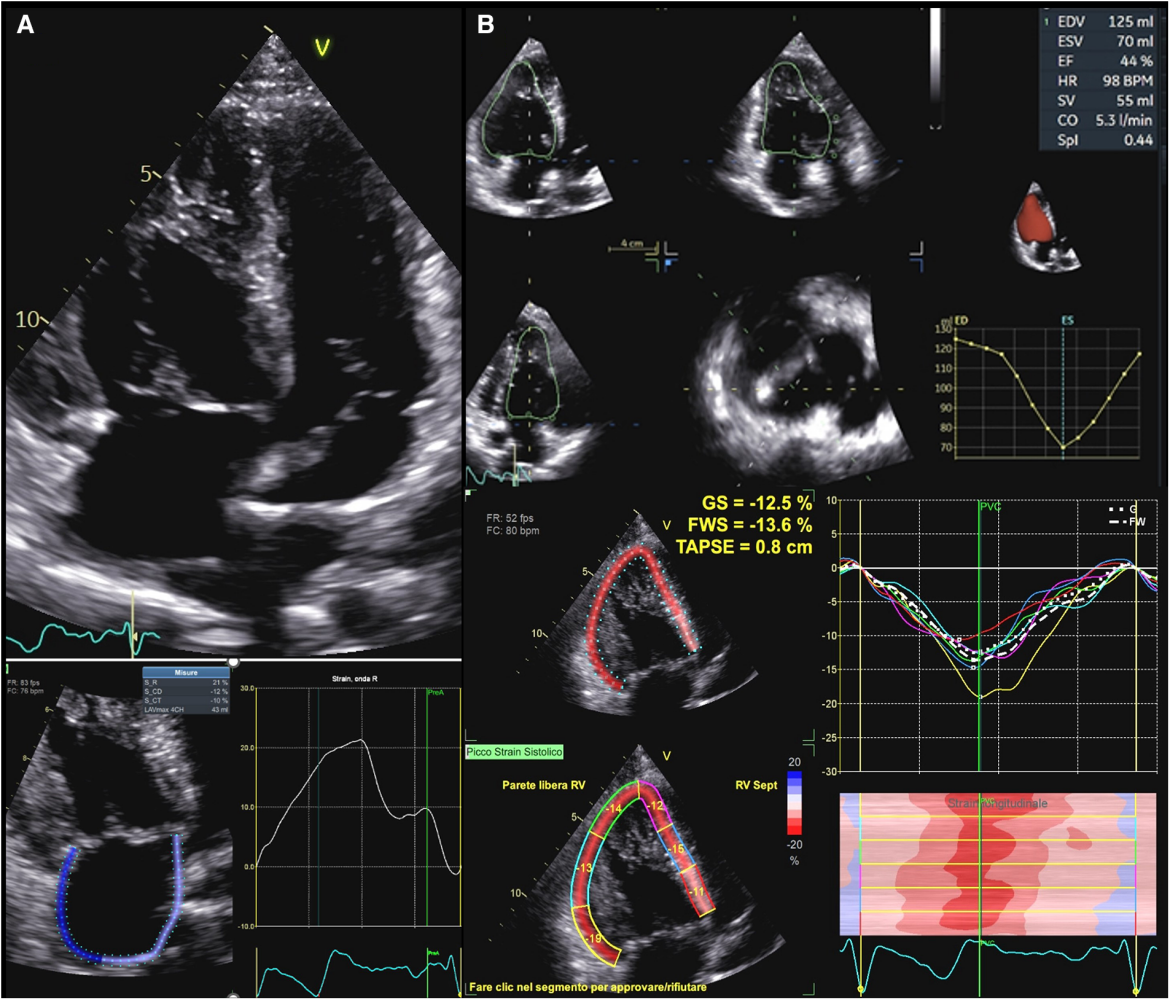
Functional assessment in systemic right ventricle physiologies. (**A**) Upper panel: apical 2D view in a patient with D-TGA after Senning operation. Lower panel: right atrial strain in the same patient. Atrial reservoir is impaired in this patient (21%). (**B**): Upper panel: sRV function as assessed by 3DE in a patient with ccTGA. Lower panel: GLS values in the same patient, which are impaired in all segments. D-TGA, D-transposition of great arteries; sRV, systemic right ventricle; 3DE, three-dimensional echocardiography; ccTGA, congenitally corrected transposition of great arteries.

Although its morphological features preclude the application of formulae based on geometrical assumptions, transthoracic echocardiography remains the main imaging modality to assess systemic right ventricular function (Figure 4). Many studies compared standard echocardiographic parameters, such as TAPSE and tissue doppler velocities of the tricuspid annulus, with EF calculated by CMR showing inconclusive results (109, 110). FAC, instead, seems to show a moderate correlation with ejection fraction from CMR (111). These indexes can therefore be more useful as controls for the same patients than as a precise quantification of myocardial function. Myocardial deformation imaging is a very attractive clinical tool for the assessment of RV systolic performance, since it provides incremental diagnostic and prognostic information over the traditional indices of RV function (112). In particular, STE allows to characterize contraction patterns separating longitudinal and circumferential components. Systemic right ventricles, in fact, seem to have a more circumferential pattern of contraction than sub-pulmonary ones. As shown by Wu et al., in D-TGA who underwent Senning or Mustard operation, longitudinal strain was reduced as well as in TOF patients if compared to healthy controls. However, circumferential strain was higher in sRVs than in TOF patients or in healthy controls and it correlated with ejection fraction at MR while longitudinal strain didn't (113). Moreover, circumferential strain seems to be the best echocardiographic predictor of exercise capacity in these patients (114). So, a possible compensatory increase in circumferential strain must always be considered when hypothesizing a dysfunction of the systemic right ventricle because longitudinal parameters might not be sensitive enough.

Tricuspid regurgitation assessment is another important part of echocardiographic evaluation of these patients. Assessing the tricuspid valve can be challenging due to congenital abnormalities, complex anatomies, and poor acoustic windows. 3D transesophageal echocardiography seems to be the best tool to assess the mechanism of regurgitation since it allows to reconstruct multiplanar anatomy (109). Real-time 3-dimensional, both transthoracic and transesophageal, may, in facts, not only overcome the problem of geometric assumptions and apical foreshortening but also provide more comprehensive assessment of contraction patterns and ventricular function (115).

#### 9.2.1 State-of-the-art and future perspective of STE to assess cardiac mechanics in CHDs

The current guidelines from the European Society of Cardiology and the American College of Cardiology/American Heart Association on adults with CHDs lack disease-specific recommendations for the application of STE, and reference values for different CHDs; also, they do not address the role of this technique in risk stratification. Similarly, the pediatric recommendations still mention STE as a novel technique but continue to focus more on structural rather than functional heart assessment (116–118).

However, much data showed the feasibility and reproducibility of this technique, which is reported to be good in different studies including patients with different CHDs (119, 120) and modest in patients with complex anatomy such as single ventricle physiologies (121, 122).

To enhance the reliability and reproducibility of strain analysis, a standardized approach is fundamental, and this follows the EACVI consensus document recommendations for adult patients (123). To optimize the analysis, the temporal resolution of image acquisition should be higher than 50 fps (124). Regulating the acquisition depth and the sector width are strategies to increase image frame rate (125). Once the images are collected, it is generally either required to trace the endocardial border or to indicate some reference points of the region of interest (ROI). The automatized tracking must always be inspected before proceeding to results: good tracking should follow the endocardial border throughout the cardiac cycle. It might be necessary to adjust the ROI width according to cardiac width, so that only cardiac walls are included. At this point, the system will provide strain values for the assessed structure: a bull eye for the left ventricle with an average global longitudinal strain, a longitudinal strain value separately for right ventricle free wall and the septum and 3 values for LA referring to reservoir, conduit and contractile function.

Data about the prognostic values of STE in CHDs are scarce. Still, a very recent meta-analysis including 33 studies found that RV GLS, LV GLS, and both, were associated with major adverse cardiovascular events in ccTGA/atrial switch, congenital aortic stenosis/bicuspid valve and TOF, respectively. Also, strain and strain rate of single ventricle showed associations with outcomes in HLHS during the interstage phase and following stage 2 and Fontan procedures, yet not preceding stage 1/Norwood (126).

This meta-analysis supports the need for an update of the current recommendations on the use of STE with specific data on CHDs and should encourage the CHD community to develop standardized protocols and reference values to enhance diagnostic precision, consistency and to impact patients' management providing information on optimal time for intervention, correction and prognosis (127).

## Conclusion

In conclusion, echocardiography plays a central role in the comprehensive assessment of cardiac mechanics in CHDs. In addition to standard echocardiographic parameters, continued advancements in technology, including deformation imaging and 3D echocardiography further enhance its effectiveness, making echocardiography an indispensable component of the multidisciplinary approach to managing and improving outcomes for individuals with congenital heart anomalies.
